# Roles of VEGF-Flt-1 signaling in malignant behaviors of oral squamous cell carcinoma

**DOI:** 10.1371/journal.pone.0187092

**Published:** 2017-11-17

**Authors:** Ajiravudh Subarnbhesaj, Mutsumi Miyauchi, Chea Chanbora, Aki Mikuriya, Phuong Thao Nguyen, Hisako Furusho, Nurina Febriyanti Ayuningtyas, Minoru Fujita, Shigeaki Toratani, Masaaki Takechi, Shumpei Niida, Takashi Takata

**Affiliations:** 1 Department of Oral and Maxillofacial Pathobiology, School of Biomedical and Health Sciences, Hiroshima University, Hiroshima, Japan; 2 Department of Global Dental Medicine and Pharmacy at Ho Chi Minh city, Integrated Health Sciences, School of Biomedical and Health Sciences, Hiroshima University, Hiroshima, Japan; 3 Department of Oral and Maxillofacial Radiology, Institute of Biomedical and Health Sciences, Hiroshima University, Hiroshima, Japan; 4 Department of Molecular Oral Medicine and Maxillofacial Surgery, Institute of Biomedical and Health Sciences, Hiroshima University, Hiroshima University, Hiroshima, Japan; 5 Department of Oral and Maxillofacial Surgery, Institute of Biomedical and Health Sciences, Hiroshima University, Hiroshima, Japan; 6 Biobank, Medical Genome Center, National Center for Geriatrics and Gerontology, Obu, Japan; Charles P. Darby Children's Research Institute, 173 Ashley Avenue, Charleston, SC 29425, USA, UNITED STATES

## Abstract

**Background:**

Vascular endothelial growth factor (VEGF) is a highly specific signaling protein for vascular endothelial cells that plays a critical role in tumor growth and invasion through angiogenesis, and may contribute to cell migration and activation of pre-osteoclasts, osteoclasts and some tumor cells.

**Objectives:**

We aimed to clarify the detailed roles of VEGF-Flt-1 signaling in bone invasion of oral squamous cell carcinoma (OSCC) cells.

**Results:**

Forty-two (42) of 54 cases with gingival SCC (77.8%) strongly expressed VEGF, and had a significantly increased number of Flt-1+ osteoclasts (p<0.01) and more aggressive bone invasion (p<0.05). PlGF, a ligand of Flt-1, induced osteoclastogenesis in single culture of bone marrow cells (BMCs), and inhibition of Flt-1-signaling by VEGF tyrosine kinase inhibitor and It’s down stream (Akt and ERK1/2) inhibitos reduced osteoclastogenesis in PlGF-stimulated BMCs (p<0.01). In molecular level, PlGF stimulation significantly upregulated RANKL expression in Flt-1-expressing HSC2 cells via phosphorylation of Akt and ERK1/2. In the co-culture of VEGF-producing HSC2 cells and BMCs, number of TRAP-positive osteoclasts markedly increased (p<0.01). The osteoclastogenesis was significantly inhibited by RANKL-neutralizing antibody (p<0.01) as well as by VEGF tyrosine kinase inhibitor (p<0.01) and it’s downstream (Akt and ERK1/2) inhibitors (p<0.01, p<0.05, respectively).

**Conclusion:**

VEGF-Flt-1 signaling induces osteoclastogenesis in OSCC through two possible ways: 1) VEGF produced from OSCC cells can directly stimulate the Flt-1 pathway in preosteoclasts to induce migration to future bone resorbing area and differentiation into osteoclasts, and 2) VEGF-Flt-1 signaling upregulates RANKL expression in OSCC cells, which indirectly leads to osteoclast differentiation. Therefore, blocking of the VEGF-Flt-1 signaling may help inhibit bone invasion of OSCC.

## Introduction

Head and neck cancers represent the sixth most common cancer worldwide; approximately 630,000 new patients are diagnosed annually, and there are more than 350,000 deaths every year [1]. Head and neck cancers are defined as a heterogeneous group of aggressive epithelial malignancies that develop from the mucosal linings in the head and neck area [2]. More than 90% of head and neck cancers are squamous cell carcinoma (SCC), which mainly occurs in the oral cavity and oropharynx, so-called oral squamous cell carcinoma (OSCC) [3,4].

Like most malignancies, OSCC has highly malignant behaviors, including invasion, recurrence and metastasis. A major problem is tumor invasion into the adjoining maxilla and mandible [5]. Gingival SCC, in particular, frequently invades into the underlying bone. This event can lead to a poor prognosis, and the treatments such as mandibulectomy, radiation and chemotherapy can tremendously reduce the quality of life of OSCC patients [6,7]. However, the cellular and molecular mechanisms regulating bone invasion by OSCC are still not well understood.

Angiogenesis is a physiological process through which new blood vessels form from pre-existing blood vessels. This process is indispensably crucial for cancer growth, progression and metastasis. It is generally known that VEGF is one of the most important proangiogenic factors [8]. VEGF is produced by multiple cell types, including macrophages and osteoblasts [9,10]. The VEGF family currently includes seven members, VEGF-A, VEGF-B, VEGF-C, VEGF-D, VEGF-E, VEGF-F, and Placental Growth Factor (PlGF) [8]. VEGF-A is well known as a key regulator of physiological angiogenesis and hematopoiesis [11, 12] and has been implicated in the establishment of epiphyseal vascularization and endochondral ossification [13, 14]. VEGF-A binds to two tyrosine kinase (TK) receptors, Flt-1 (fms-like tyrosine kinase receptor 1) and Flk-1 (fetal liver kinase 1), which serve as key mediators for angiogenesis [15–17]. Masood et al. [17] reported the concurrent expression of VEGF and its receptors in a number of tumor cells and suggested that VEGF functions as an autocrine growth factor. It is well accepted that the activation of Flt-1 by VEGF induces cell migration. Flt-1 is expressed in monocytes and regulates their activation and chemotaxis [18,19]. Interestingly, monocyte/macrophage lineage cells including osteoclasts were reported to express Flt-1 [20,21]. There is support that Flt-1 might be involved in osteoclastogenesis. However, its direct roles in bone invasion and other malignant behaviors of OSCC are still not well understood.

In the present study, we aimed to clarify the correlation between VEGF expression and the severity of bone invasion in gingival SCC, and we examined the effect of OSCC-produced VEGF on osteoclastogenesis. Furthermore, the mechanism and signal transduction of VEGF, which induces osteoclastogenesis, were investigated at the molecular level.

## Materials and methods

### Patient specimens

Fifty-five cases of gingival SCC were retrieved from the pathological files of Hiroshima University Hospital, Japan. All cases involved first operation specimens, including the interface between the resorbing bone margin and OSCC. Clinical details including the patient age, sex, tumor location, tumor size and degree of bone invasion were gathered from surgical records of the patients (24 males and 30 females; age 69.4 ± 11.5 years; 23 maxillas and 31 mandibles). The study was approved by the ethnical committee of Hiroshima University (Permit Number: 1237).

To evaluate the degree of bone destruction on radiography, the radiographic appearances of the tumor were graded into 3 grades (S1 Fig): Grade 1—No bone resorption or only erosion on the superficial surface; Grade 2—Bone resorption observed within the alveolar bone; and Grade 3—Extensive bone resorption involving the inferior alveolar canal or maxillary sinus floor, basically according to the TNM clinical classification of the primary tumour [22].

#### Immunohistochemistry

Unstained 4.5-μm sections were de-paraffinized and rehydrated by routine histological techniques. Endogenous peroxidase activity was blocked with 0.3% H_2_O_2_ in methanol for 30 minutes. The sections were then incubated with protein block serum-free solution (DAKO, Japan) for 10 minutes. VEGF polyclonal antibody (V-3, IBL, Japan) and Flt-1 polyclonal antibody (H-225, Santa Cruz Biotechnology Inc., USA) were diluted in sterile PBS (1:50 and 1:100, respectively) and incubated overnight at 4°C. The sections were incubated with labeled polymer-HRP-anti-rabbit (DAKO) for 1 hour at room temperature. The color was developed with 0.025% 3,3’-diaminobenzidine tetrahydrochloride in Tris-HCl buffer plus hydrogen peroxide (DAB; DAKO).

Evaluation of VEGF immunostaining was based on the proportion of stained cancer cells and divided into 4 grades, which were Grade I—cancer cells were completely negative, Grade II—fewer than 30% of cancer cells were positively stained, Grade III—30–70% of cancer cells were positively stained, and Grade IV—more than 70% of cancer cells were positively stained. Grades I and II were grouped as “Low expression,” and Grades III and IV were grouped as “High expression.”

Osteoclasts along the bone margin at the tumor/bone interface (S2A Fig) were positively stained with Flt-1 (S2B Fig). The 100x magnification photograph was taken at tumor/bone interface. The number of Flt-1+osteoclasts along the bone surface was counted on the photograph (unit area of 0.67mm^2^). OSCC cases were classified into 2 groups: low grade osteoclast number group contained 0–5 Flt-1+osteoclasts, and the high grade osteoclast number group contained >6 Flt-1+osteoclasts.

### Single and co-culture experiments

This study was carried out in strict accordance with the recommendations in the Guide for the Care and Use of Laboratory Animals of the Hiroshima University Animal Research Committee and AVMA Guidelines on Euthanasia. The protocol described below was approved by the Committee on the Ethics of Animal Experiments of the Hiroshima University (Permit Number: A11-141). All mice were housed in a specific pathogen free facility in 12 hr light-dark cycles with access to water and food ad libitum. Under Carbon dioxide inhalation, the tibiae and femurs were obtained from 5-6-week-old C57BL/6 male mice. And bone marrow cells were isolated. Then, 10^5^ cells per well in a 96-well plate were cultured in α-MEM containing murine M-CSF 20 ng/ml, supplemented with 10% heat-inactivated FBS (Invitrogen, USA) and 100 U/ml penicillin-streptomycin (Invitrogen) under conditions of 5% CO_2_ at 37°C. After 2 days, adherent cells were used as bone-marrow-derived monocyte/macrophage precursor cells (BMCs) after washing out the non-adherent cells including lymphocytes.

For the single culture experiment, the BMCs were continuously maintained with 20 ng/ml m-MCSF and treated with Flt-1-specific ligand (PlGF) (10 ng/ml) (Peprotech, USA) with or without VEGFR tyrosin kinase inhibitor (VRI: CALBIOCHEM, Germany) (10 μM), an inhibitor PI3 kinase-dependent Akt phosphorylation and kinase activity (LY294002; Sigma-Aldrich, USA) (10 μM), ERK inhibitor (UO126; Sigma-Aldrich, USA) (10 μM) or RANKL-neutralizing antibody (eBioscience) (0.5 μg/ml). The single culture of BMCs with recombinant RANKL (peprotech, USA) (100ng/ml) stimulation was used as a positive control.

For pit formation assay, trypsinized BMCs were plated on dentin slices in 96-well culture plates and cultured for 1 hour according to the Nakayama’s method [23]. The dentin slice were then transferred into 48-well culture plates and culture in α-MEM containing PlGF with or without LY294002, U0126, VRI or RANKL-neutralizing antibody. RANKL stimulated BMC culture on dentin slice also done as appositive control.

For the co-culture experiment, the BMCs with 20 ng/ml m-MCSF were cultured with HSC2 cells with or without LY294002, U0126, VRI or RANKL-neutralizing antibody in a 1:1 mixed medium of RPMI 1640 (Nissui Pharmaceutical Co., Japan) and α-MEM. The BMCs were also co-cultured with PlGF-pretreated HSC2 cells in a 1:1 mixed medium of RPMI 1640 and α-MEM as positive control.

After 3 days, cells were stained with tartrate-resistant acid phosphatase (TRAP) activity according to the method of Minkin [24]. All TRAP-positive multinuclear cells (>3 nuclei) in each well were counted as osteoclasts. The results were expressed as averages with the standard error. All assays were performed in triplicate.

### Cell culture

Six OSCC cell lines (HSC2, HSC3, HSC4, Ca9-22, Ho-1-N-1, and Ho-1-U1), which were used for this study, were provided by the Japanese Collection of Research Bioresources Cell Bank. Cells were grown in RPMI 1640 supplemented with 10% FBS at 37°C and 5% CO_2_.

### RT-PCR analysis

The HSC2 cells were stimulated by 10ng/ml of PlGF with or without LY294002, U0126 or VRI. Total RNA was extracted using the RNeasy Mini Kit (Qiagen, K.K., Tokyo, Japan) according to the manufacturer’s instructions. The RNA concentration and purity were determined using standard spectrophotometric methods. One microgram of total RNA was used for cDNA synthesis with a ReverTra Dash Kit (Toyobo, Osaka, Japan). Total cDNA was amplified using Go Taq Green Master Mix (Promega, Madison, WI, USA). Amplification of human RANKL and GAPDH was performed in a MyCyclerTM thermal cycler (Bio-Rad, Tokyo, Japan) for 30 cycles with denaturation for 30 s at 94°C, annealing for 30 s at 58°C, and extension for 1 min at 72°C and the primers for each. The amplification products were resolved on 1.5% agarose/TAE gels (Nacalai Tesque, Inc., Kyoto, Japan), electrophoresed at 100 mV, and visualized by ethidium bromide staining. The primer pair sequences are Forward, 5’-CTGCCATCATCTTTGGCGTTTG-3’, Reverse, 5’- GTTCAGAGAAAGGAGGTGTGGA-3’ for RANKL; and Forward, 5’-ACAGTCAGCCGCATCTTCTT-3’, Reverse, 5’-TTGATTTTGGAGGGATCTCG-3’ for GAPDH.

### Western blot analysis

HSC2 cells were treated with 10ng/ml PlGF and harvested at indicated time to analyze PlGF signaling. The cells were lysed in ice–cold lysis buffer containing 50 mM Tris-HCl (pH 7.5), 250 mM NaCl, 0.1% Triton X-100 (Roche, Castle Hill, Australia), 1 mM EDTA, 50 mM NaF, 0.1 mM Na_3_VO_4_, 1 mM DTT, 0.1 mM leupeptin, 0.1 μg/ml soybean trypsin inhibitor, 10 μg/ml L-1 chlor-3-(4-tosylamido)-4-phenyl-2-butanon (TPCK), 10 μg/ml L-1 chlor-3-(4-tosylamido)-7-amino-2-heptanon-hydrochloride (TLCK), 10 μg/ml aprotinin and 50 μg/ml phenylmethylsulfonyl fluoride (PMSF). Lysates were incubated on ice for 30 minutes and centrifuged at 13,400 rpm for 20 min at 4°C. Supernatants were collected as a whole lysate. The protein concentration was determined by the Bradford protein assay (Bio-Rad, USA) using bovine serum albumin (Sigma) as a standard. Then, 25 μg of protein was subjected to 10% polyacrylamide gel electrophoresis followed by electroblotting onto a nitrocellulose filter. Primary and secondary antibodies were applied as the datasheet indicated. For detection of the immune complex, the ECL western blotting detection system (Amersham Biosciences, UK) was used.

The following antibodies, obtained from Cell Signaling, were used: p-Akt (9271; diluted to 1:1000), anti-total-Akt (4691; diluted to 1:1000), anti-phospho-ERK1/2 (4376; diluted to 1:1000), and anti-total-ERK1/2 (4695; diluted to 1:1000). Anti-VEGF (18413; IBL, Japan; 2 μg/ml), Anti-Flt-1 (sc316; Santa Cruz Biotechnology, USA; diluted to 1:500) and β-actin (A2228; Sigma-Aldrich; diluted to 1:8000) was also employed.

### Statistical analysis

SSRI for Windows (Social Survey Research Information Co., Ltd., Tokyo, Japan) was used for statistical analysis. The experiments were performed three times. The statistical significance of the cross-tabulation table regarding the possible correlation between the VEGF expression level and osteoclast number/bone destruction was analyzed by the chi-square test. *p*-values < 0.05 were considered statistically significant. The correlation between variables in *in vitro* studies was analyzed by Student’s *t*-test. The data are presented as the mean ± standard deviation (SD). Two side *p*-values < 0.05 were considered statistically significant.

## Results

### VEGF expression in gingival SCC correlates with aggressive bone invasion

**Fig 1:**
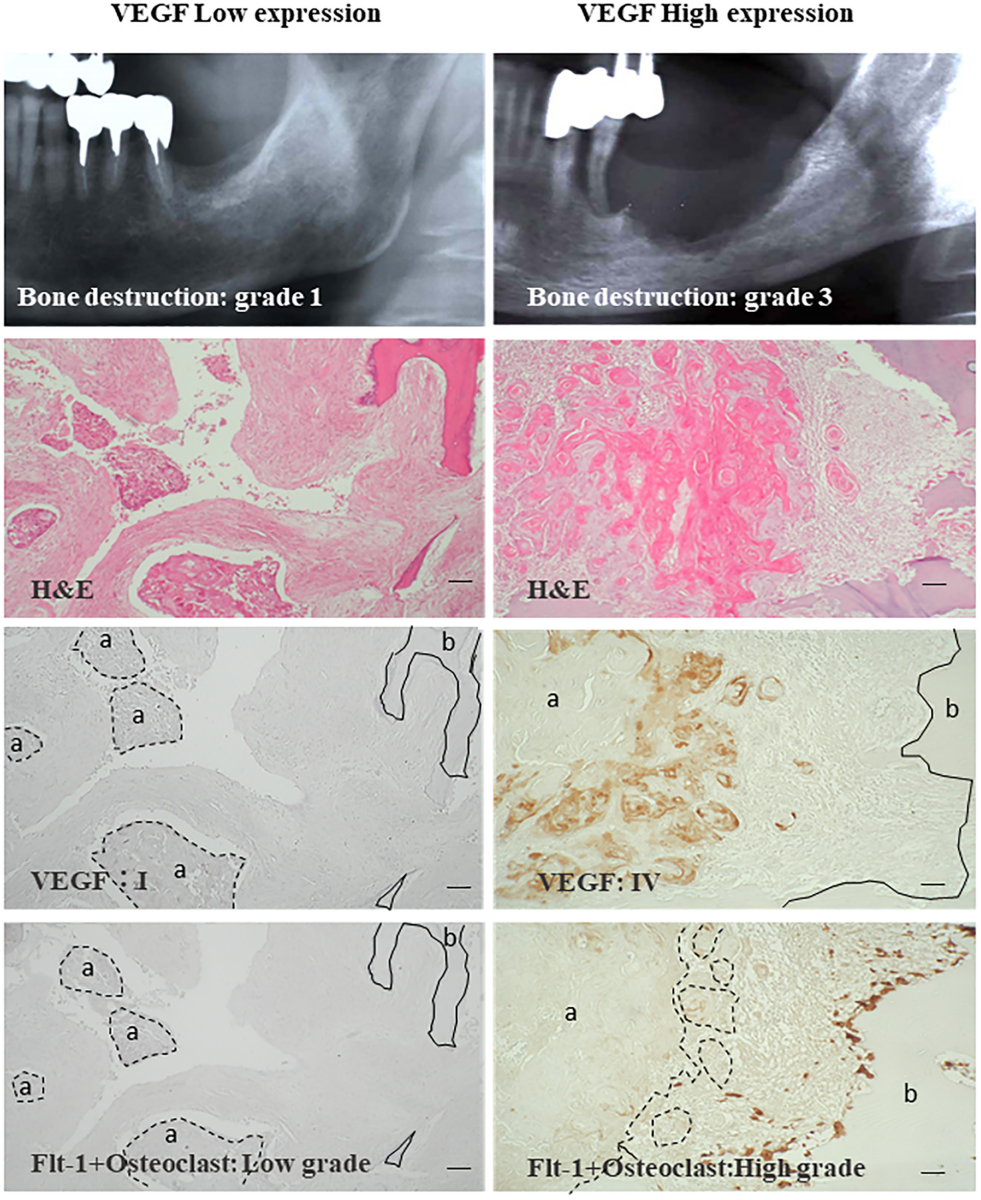
Representative radiographic and histologic appearances of the VEGF low and high expression cases. (A) A representative case from the VEGF low expression group (N = 18). Bone destruction appears as superficial surface erosion. The tumor area is not or is weakly positive for VEGF, and Flt-1+ osteoclasts are rarely seen at the tumor/bone interface. (B) A representative case of the VEGF high expression group (N = 37). OSCC cells are strongly positive for VEGF, and numerous Flt-1+osteoclasts are seen at the outer surface of resorbing bone margin. Scale bar = 100 μm. dot line area (a): tumor nest, solid line area (b): bone.

To evaluate the effect of OSCC-produced VEGF on OSCC bone invasion, we immunohistochemically analyzed VEGF expression in OSCC cells and Flt-1+ osteoclast numbers at the bone invasion front in 54 cases of gingival SCC by comparison with the degree of bone invasion on radiography. Forty-two of 54 cases (77.8%) strongly expressed VEGF (VEGF high expression), while 12 cases (22.2%) showed low VEGF expression. Fig 1 shows representative cases of VEGF low and high expression with Flt-1 expression and radiography. Numerous Flt-1+osteoclasts were observed along the bone resorbing margin in the VEGF high expression cases where there was bone destruction beyond the mandibular canal, which was radiographically seen in Grade 3 cases. However, the number of osteoclasts was low in the low VEGF expression cases. Radiographic examination only showed erosion on the superficial surface of alveolar bone, indicating Grade 1.

**Fig 2:**
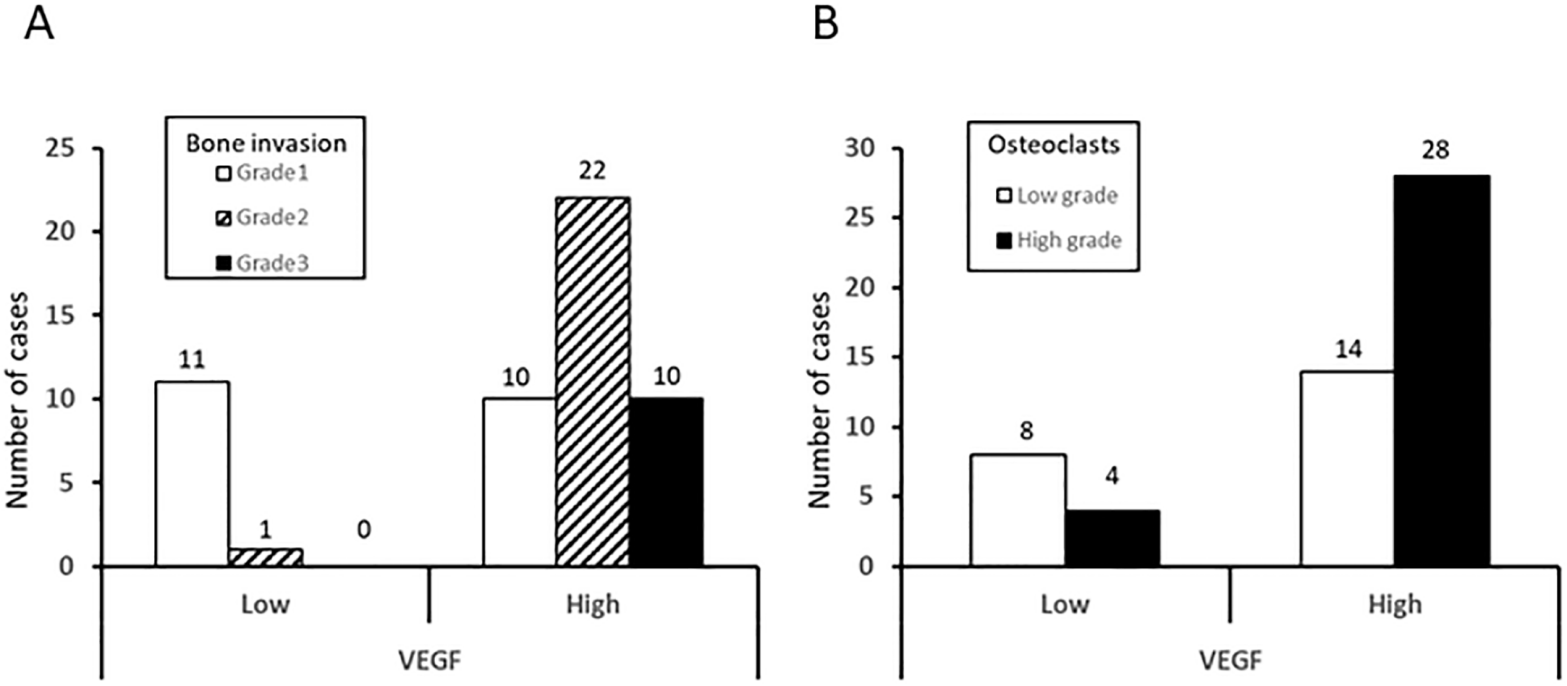
Relationship between VEGF expression and bone invasion and relationship between VEGF expression and the Flt-1-expressing osteoclast number. (A) There were 21 Grade 1 bone invasion cases, 23 of Grade 2 and 10 of Grade 3. The high VEGF expression group has a significantly more aggressive radiographic pattern of bone invasion. the chi-square test; p<0.05 (B) Flt-1+osteoclasts along the irregular bone margin were counted. Twenty-two in the low grade of osteoclasts number and 32 in the high grade are included. The number of Flt-1+ osteoclasts in the high VEGF expression group is higher than that in the low VEGF expression group. the chi-square test; p<0.01.

There were 21 Grade 1 bone invasion cases, 23 of Grade 2 and 10 of Grade 3. VEGF-low expression group included 11 cases of Grade 1 and 1 cases of Grade 2. While VEGF high expression group contained 10 cases of Grade 1, 22 cases of Grade 2 and 10 cases of Grade 3. The VEGF high expression cases had a significantly more aggressive radiographic pattern of bone invasion (p<0.01) (Fig 2A). Fig 2B showed that VEGF high expression cases has a significantly increased number of Flt-1+ osteoclasts (p<0.05).

### OSCC-produced VEGF directly induces osteoclastogenesis by stimulating Flt-1+ preosteoclasts

**Fig 3:**
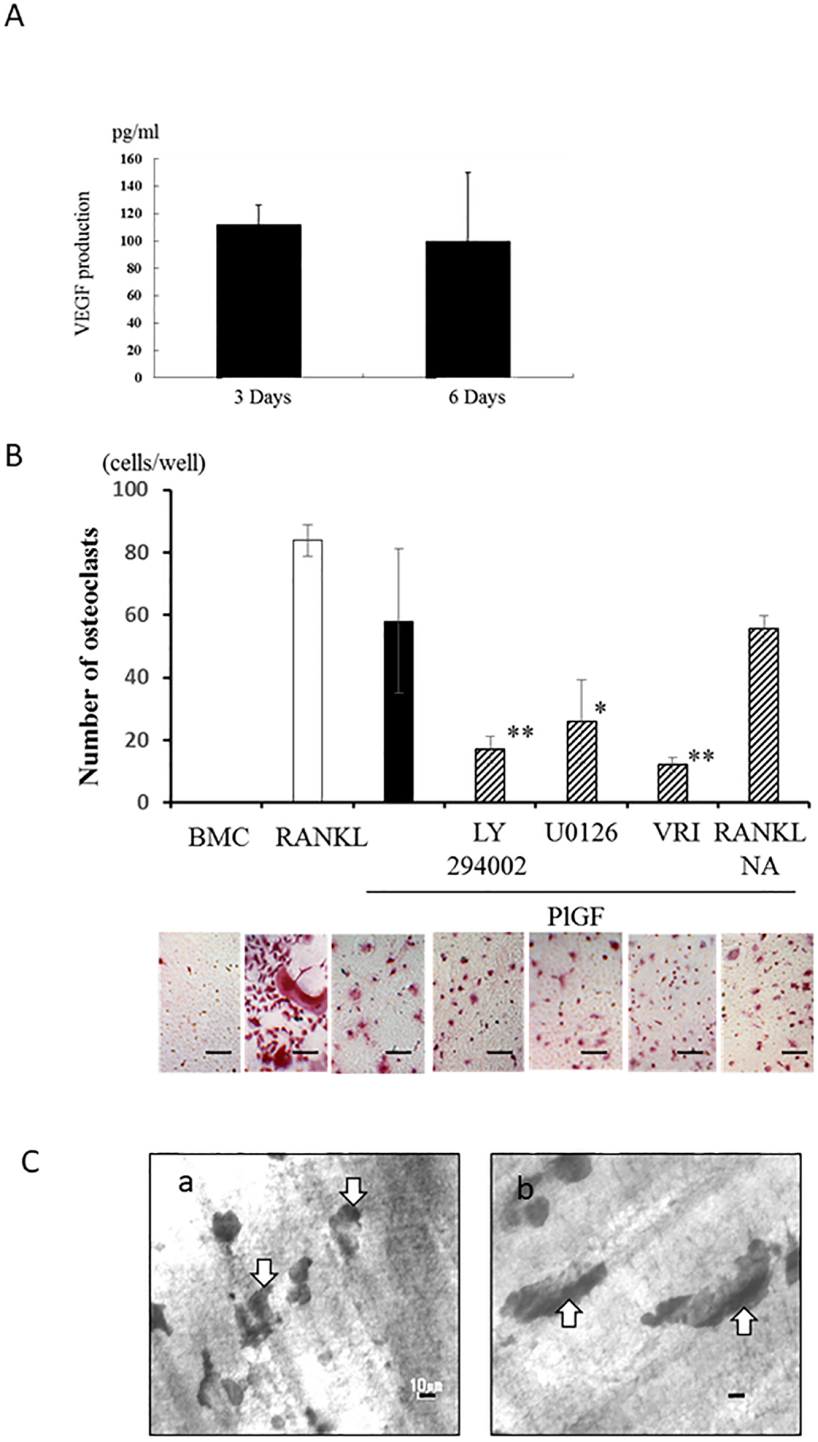
The direct role of VEGF-Flt-1 signaling in osteoclastogenesis caused by OSCC. (A) The VEGF-A production in culture medium of HSC2 cells was measured by ELISA at 3 and 6 days. HSC2 cells constitutively produced VEGF-A. (B) Single culture of bone marrow cells (BMCs) treated with RANKL (100 ng/ml) or Flt-1-specific ligand (placental growth factor; PlGF (10 ng/ml)) with/without Akt inhibiter (LY29400: 10 μM), ERK inhibitor (U0126: 10 μM), VEGF tyrosine kinase inhibitor II (VRI: 10 μM) or RANKL-neutralizing antibody (5 μg/ml) was performed in the presence of mMCSF (20 ng/ml). After 3 days, TRAP-positive osteoclasts were counted. PlGF, similar to RANKL, has a significant stimulatory effect on osteoclastogenesis. Scale bar = 50 μm. (C) Bone resorbing activity of PlGF-induced osteoclasts was confirmed by pit formation assay using dentin slice. (a) Pits (white arrows) made by PlGF-induced osteoclasts. (b) Pits (white arrows) made by RANKL-induced osteoclasts. Scale bar = 10 μm.

BMCs can differentiate into osteoclasts in the presence of RANKL and M-CSF, and VEGF injection induced osteoclasts in M-CSF deficient op/op mouse, indicating VEGF can substitute M-CSF. Moreover, Flt-1 is a major receptor for monocyte/osteoclast lineage cell migration and osteoclastogenesis [20, 21]. VEGF-A production in culture media from HSC2 cells has been confirmed by ELISA (Fig 3A). To clarify the role of VEGF-Flt-1 signaling in osteoclast-stimulating activity of OSCCs, we performed a further experiment using a single culture system for BMCs and co-culture system for BMCs and HSC2 cells.

PlGF is well known as a ligand of Flt-1 but not Flk-1; therefore, we performed the single culture of BMCs stimulated with PlGF. PlGF had a significant stimulatory effect on osteoclastogenesis like RANKL. PlGF-induced osteoclasts were smaller than RANKL-induced osteoclasts. Pit formation assay showed that PlGF-induced osteoclast resorbed dentin slice and made smaller pits comparing to those by RANKL-induced osteoclasts (Fig 3C). The use of VRI (p<0.01), Akt inhibitor (p<0.01) and ERK1/2 inhibitor (p<0.05) significantly inhibited the PlGF-induced osteoclastogenesis (Fig 3B). VRI and Akt inhibitor also significantly reduced the number of resorbing pits (S4 Fig). On the other hand, RANKL-neutralizing antibody had no effect on PlGF-induced osteoclastogenesis (Fig 3B and S4 Fig), indicating that PlGF directly induced osteoclast differentiation without RANKL production.

### VEGF indirectly induces osteoclastogenesis by upregulating RANKL expression in OSCC cells via VEGF-Flt-1 signaling

Since we observed Flt-1 immunolocalization in preosteoclasts, osteoclasts and some OSCC cells (S3 Fig), we firstly aimed to clarify the role of VEGF-Flt-1 signaling in OSCC cells at the molecular level.

**Fig 4:**
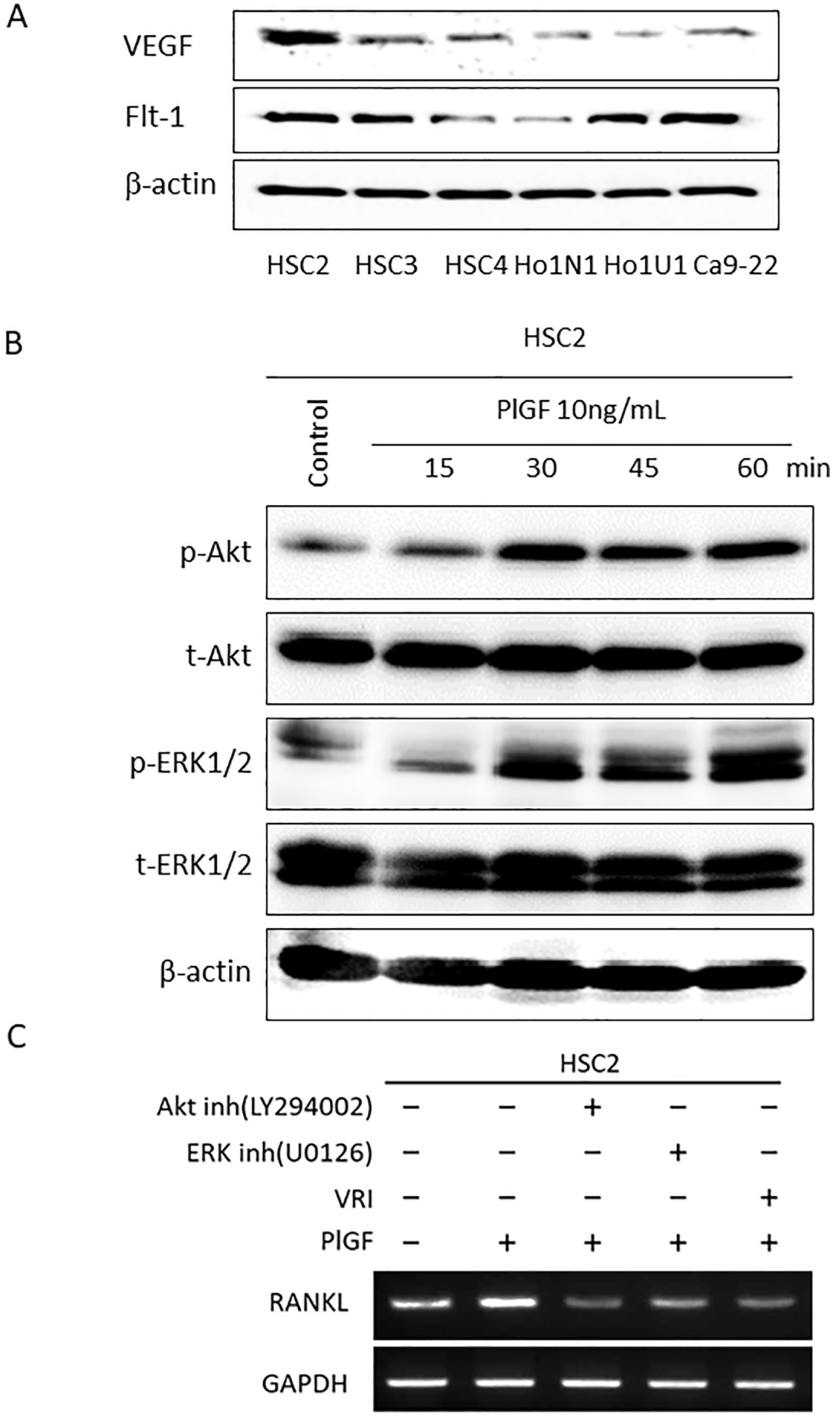
The role of VEGF-Flt-1 signaling in RANKL expression of OSCC. (A) VEGF and Flt-1 expression in several OSCC cell lines was examined by western blot analysis. (B) 10 ng/ml of PlGF was applied to HSC2 cells. After 0, 15, 30, 45 and 60 minutes, phosphorylation of Akt and ERK1/2 was examined by western blot analysis. (C) HSC2 cells were stimulated by PlGF (10 ng/ml) combined with Akt inhibitor (LY294002; 10 μM), ERK inhibitor (U0126; 10 μM) or VRI (10 μM). After 3 days, the RANKL mRNA expression was examined by RT-PCR.

Fig 4A shows the expression of VEGF and Flt-1 in 6 OSCC cell lines at various levels. HSC2 cells expressed a high level of VEGF and Flt-1 among the 6 OSCC cell lines. Therefore, we investigated the effect of VEGF-Flt-1 signaling on RANKL expression in HSC2 cells. VEGF-Flt-1 signaling activation by PlGF induced upregulation of RANKL expression in HSC2 cells (Fig 4C). Next, we clarified the signaling pathways involved in the PlGF-induced RANKL expression. PlGF activated phosphorylation of Akt and ERK1/2 in HSC2 cells (Fig 4B). To identify the intracellular signaling pathway mediating PlGF-induced RANKL expression, HSC2 cells were preincubated with Akt, ERK inhibitors or VRI for 30 minutes and then incubated with PlGF for 3 days. In addition to VRI, Akt and ERK inhibitors markedly downregulated PlGF-induced RANKL expression in HSC2 cells (Fig 4C).

**Fig 5:**
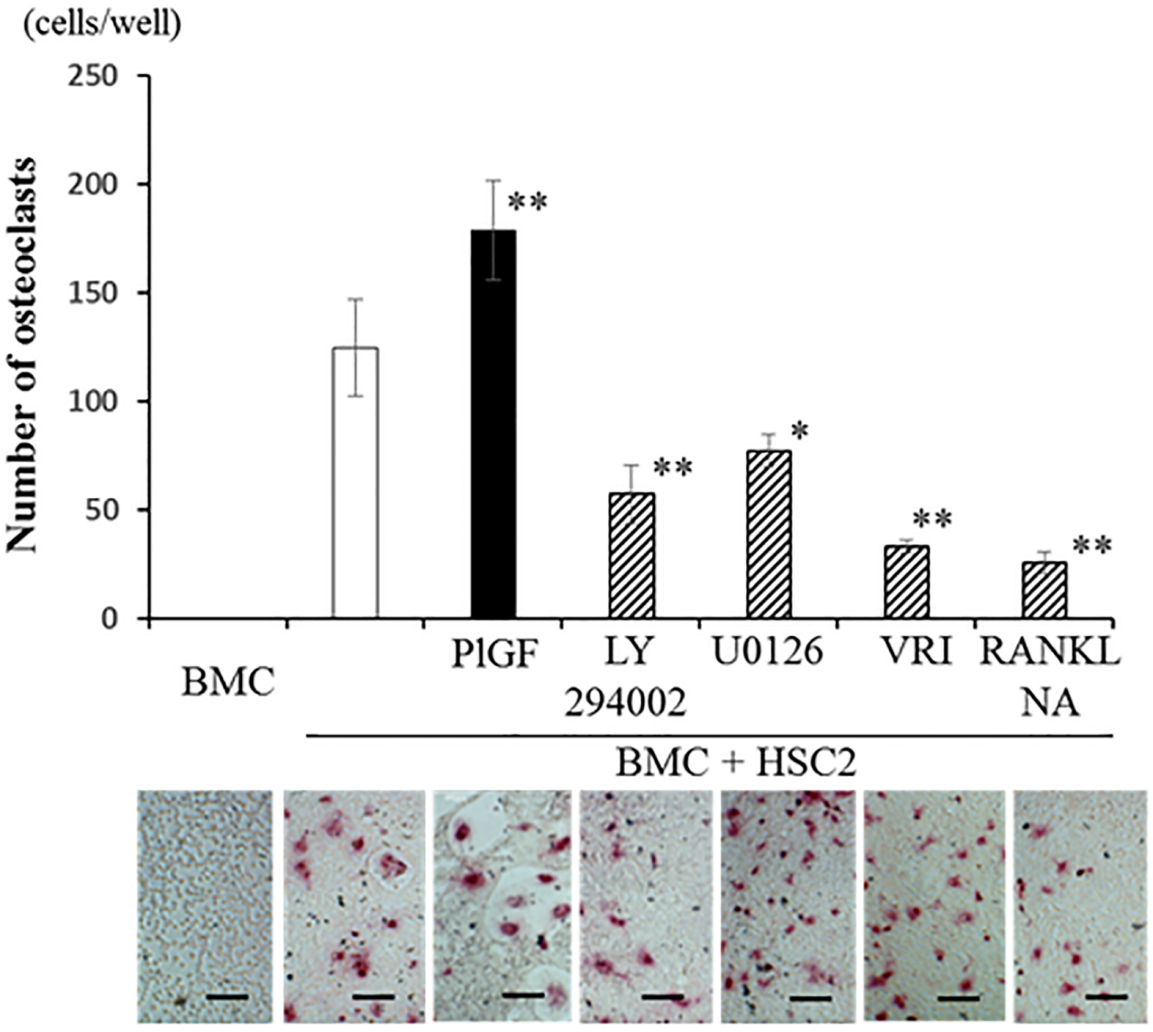
The indirect role of VEGF-Flt-1 signaling in osteoclastogenesis caused by OSCC. Co-culture of VEGF-producing OSCC cells (HSC2) and BMC with or without Akt inhibitor (LY29400: 10μM), ERK inhibitor (U0126; 10 μM), VEGF tyrosine kinase inhibitor II (VRI; 10 μM) or RANKL-neutralizing antibody (0.5 μg/ml) treatment was performed in the presence of mMCSF (20 ng/ml). After 3 days, TRAP-positive osteoclasts were counted. *p<0.05, **p<0.01. Scale bar = 50 μM.

Next, to confirm the importance of indirect pathway of osteoclastogenesis through RANKL expression in OSCC cells caused by VEGF-Flt-1 signaling, we examined the effects of signal transduction inhibitors using co-culture of HSC2 cells and BMC. Co-culture of HSC2 cells and BMC induced numerous TRAP-positive osteoclasts (p<0.01). Activation of VEGF-Flt-1 signaling with PlGF significantly increased osteoclasts (p<0.01), which were lager in size comparing to PlGF-induced osteoclasts in BMC single culture system. VRI (p<0.01), Akt (p<0.01) and ERK (p<0.05) inhibitors and RANKL-neutralizing antibody (p<0.01) significantly downregulated osteoclastogenesis in the co-culture of BMCs and HSC2 cells (Fig 5).

## Discussion

Vascular endothelial growth factor (VEGF) is the most important cytokine that induces vascular angiogenesis in physiologic conditions and in tumor angiogenesis. It is well accepted that VEGF promotes tumor growth and metastasis of various solid tumors through tumor angiogenesis [25, 26]. VEGF binds to two tyrosine kinase receptors, Flt-1 and Flk-1. The Flt-1 and Flk-1 receptors are predominantly expressed in endothelial cells. Other cell types including osteoblasts, monocytes and macrophages also express both VEGF receptors. The signal transduction cascades induced by Flt-1 and Flk-1 are somewhat different. Flt-1 signaling mainly promotes the migration of endothelial cells and monocytes/macrophages, but the stimulatory effect on cell proliferation is weak. While Flk-1 mainly contributes to endothelial cell growth, survival and vascular permeability [21,27]. Similarly, preosteoclasts and osteoclasts express Flt-1 and Flk-1. Moreover, it is reported that Flt-1 is one of the functional receptors in osteoclasts, and mainly contributes to osteoclast migration and differentiation, while Flk-1 is known to be involved in the survival of osteoclasts [28]. Therefore, we focused on the importance of VEGF-Flt-1 signaling in bone destruction and bone invasion caused by OSCC.

To determine the role of VEGF-Flt-1 signaling in the bone destruction associated with OSCC, we immunohistochemically evaluated VEGF expression in OSCC cells and Flt-1+osteoclast numbers at the bone invasion front in 54 gingival SCC cases. Then relationship between VEGF expression and Flt-1+osteoclasts number or the degree of bone invasion on radiography were examined. VEGF expression in OSCC cases significantly correlated with a more advanced degree of bone destruction on radiographs and a higher number of Flt-1+osteoclasts at the tumor/bone interface. Therefore, it is suggested that VEGF expression in OSCC cells is an indicator of the severity of bone invasion in gingival SCC. Recent studies have established that bone resorption by osteoclasts is an important step in the process of bone invasion and metastasis in several types of malignancies [29]. Osteoclasts, which are formed by the fusion of mononuclear preosteoclasts derived from monocyte-macrophage lineage cells, are primarily responsible for tumor-induced bone destruction [30, 31]. Aldridge et al. reported that VEGF induced monocyte precursors to differentiate into osteoclasts and suggested that VEGF was important osteolytic factor in breast cancer metastases to bone [32].

To determine the effect of VEGF-Flt-1 signaling on osteoclastogenesis, murine primary bone marrow cells (BMCs) were cultured with PlGF, which can only bind to Flt-1, with or without VRI (VEGF receptor tyrosine kinase inhibitor). PlGF could induce osteoclasts from BMCs in the absence of RANKL. Application of VRI significantly reduced the number of osteoclasts caused by PlGF. And RANKL-neutralizing antibody had no effect on PlGF-induced osteoclastogenesis in single culture of BMC. These events suggested that VEGF-Flt-1 signaling in BMCs could directly contribute to osteoclastogenesis independently of RANKL. Niida et al. [20] demonstrated that VEGF can stimulate osteoclastic bone resorption *in vivo*. Moreover, to determine the function of the VEGF–Flt-1 system in osteoclast development and activity, Niida et al [33] also introduced a Flt-1-TK domain-deficient mutation (*Flt1TK*-*/*-) into *op/op* mice. The double mutant *op/op Flt1TK*-*/*- mice had an extensive osteoclast deficiency compared with *op/op* mice. Studies of bone resorption by mature osteoclasts suggest that VEGF is involved in osteoclastic recruitment and differentiation as well as in enhancing osteoclastic bone resorbing activity [34]. In the present study, although PlGF-induced osteoclasts showed bone resorbing activity, cell size was small and formed pits were limited. Henriksen et al revealed that VEGF-Flk-1 signaling induced osteoclast chemotaxis via ERK1/2 activation, while RANKL-RANK signaling activated not only chemotaxis via ERK1/2 but also bone resorption through ERK1/2-independent pathway [35]. Therefore, it is suggested that direct Flt-1-stimulation by VEGF mainly contributed to osteoclast formation and osteoclast recruitment into the bone resorption site and that additional stimulation by RANKL is needed for completely active bone resorption.

Interestingly, in this study, Flt-1 expression was observed by immunohistochemistry in preosteoclasts, osteoclasts, and many cases of OSCC. Deyama et al. [36] reported that the bone-invasive oral cancer cell line, BHY expressed detectable VEGF mRNA and VEGF induced TRAP-positive osteoclasts from BMC. In the present study, we confirmed that activation of VEGF-Flt-1 signaling upregulated RANKL expression in HSC2 cells through the Akt and ERK pathway. The differentiation of osteoclasts is mainly regulated by receptor activator of NF-kB ligand (RANKL) produced from osteoblasts [37]. Guan et al described that VEGF upregulated RANKL expression in osteoblasts, bone marrow stromal cells, leading to osteoclast activation [38]. In the primary human monocytes, VEGF-Flt-1 signaling induced chemotaxis through activation of Akt. P38 and ERK 1/2 [39]. Nakai et al. reported that mechanical stress induced bone resorption by upregulating RANKL expression via the VEGF autocrine pathway in MC3T3-E1 osteoblasts [40]. The evidences supported that VEGF stimulated Flt-1-expressing OSCC to produce RANKL in VEGF-Flt-1 autocrine pathway, which indirectly induced osteoclastogenesis at the bone invasion front.

Furthermore, we examined the effect of VEGF produced from OSCC with/without VEGF-Flt-1 signaling inhibitors using a co-culture system of BMC and HSC2 cells, which can produce VEGF. Inhibition of VEGF signaling by VRI, Akt inhibitor and ERK inhibitor could significantly suppress HSC2-induced osteoclastogenesis. Moreover, RANKL-neutralizing antibody also significantly reduced osteoclast formation, indicating the importance of RANKL upregulation in VEGF activated HSC2 cells in this process. Therefore, we considered that VEGF produced from OSCC might stimulate osteoclast differentiation at the tumor/bone interface, activating bone resorption through RANKL upregulation.

Some tumor cells can express Flt-1 or Flk-1, such as malignant melanoma [41], breast cancer [42] and colorectal cancer [43]. Masood et al. [17] reported the concurrent expression of VEGF and VEGF receptors in several tumor cells and suggested that VEGF functioned as an autocrine growth factor. It is generally accepted that the binding ability of Flt-1 with VEGF-A is 10 times higher than that of Flk-1, while the tyrosine kinase activity and self-phosphorylation of Flk-1 are stronger than those of Flt-1 [15]. Flk-1 is well accepted as the major mediator of essential functions in tumor angiogenesis, while Flt-1 may contribute to tumor growth and metastasis through recruitment/activation of macrophages [12,16]. There are several controversial studies on the expression of VEGF and VEGF receptors in OSCC [44–46]. Recently, Pianka et al. [47] analyzed VEGFR isoform immunoexpression in 50 OSCCs and confirmed that VEGF-R overexpression occurs frequently in OSCC, which might be related to the tumor size, neck node metastasis and tumor-related death. However, the role of signaling of VEGF-VEGFRs including Flt-1 in OSCC is still not completely understood and merits further study.

## Conclusions

Our findings support that VEGF-Flt-1 signaling is important in the facilitation of bone destruction and bone invasion of gingival OSCCs. There are two possible pathways in osteoclastogenesis caused by VEGF producing OSCCs; direct pathway and indirect pathway. Directly, VEGF produced from OSCC activates osteoclastogenesis through the Flt-1 pathway in preosteoclasts, inducing recruitment of osteoclasts to future resorbing bone area. Indirectly, VEGF produced from OSCC upregulates RANKL expression of OSCC in an autocrine manner through the Akt and ERK1/2 pathway, which then stimulates osteoclastogenesis. In vivo environment, RANKL-expressing OSCC can further activate VEGF-induced small osteoclasts, which migrated in bone resorbing area. Bone destruction accelerated by VEGF-Flt-1 signaling allows bone invasion of OSCC. Therefore, blocking VEGF-Flt-1 signaling may help inhibit bone invasion of OSCC.

## Supporting information

S1 Fig Radiographic grading for bone destruction by OSCC. Grade I: No bone resorption or only bone erosion on the superficial surface. Grade II: Bone resorption observed within the alveolar bone. Grade III: Bone resorption involving inferior alveolar nerve / floor of maxillary sinus.
(TIF)

S2 Fig Histological findings of the tumor-bone interface. (A) Osteoclasts are seen along the irregular bone margin at the tumor/bone interface. H&E staining. Scale bar = 100 μm. (B) Osteoclasts are positively stained with Flt-1. Immunohistochemistry, Scale bar = 10 μm.
(TIF)

S3 Fig Immunoexpression of Flt-1 at the interface between OSCC and bone. Flt-1-positive reaction was seen in osteoblasts/preosteoblasts along the bone surface as well as in OSCC cells. Immunohistochemistry, Scale bar = 100 μm.
(TIF)

S4 Fig Pit formation assay of PlGF-induced osteoclasts. Trypsinized BMCs were plated on dentin slices in 96-well culture plates and cultured for 1 hour. The dentin slice were then transferred into 48-well culture plates and culture in α-MEM containing Flt-1-specific ligand (PlGF (10 ng/ml)) with/without Akt inhibitor (LY29400: 10 μM), ERK inhibitor (U0126: 10 μM), VEGF tyrosine kinase inhibitor II (VRI: 10 μM) or RANKL-neutralizing antibody (5 μg/ml) was performed. RANKL stimulated BMC culture on dentin slice also done as a positive control. *p<0.05, **p<0.01.
(TIF)
