# Supplementary figures and images for: Roles of VEGF-Flt-1 signaling in malignant behaviors of oral squamous cell carcinoma
Source: PLoS One. 2017 Nov 17;12(11):e0187092. doi: 10.1371/journal.pone.0187092 (PMC5693288; doi:10.1371/journal.pone.0187092)

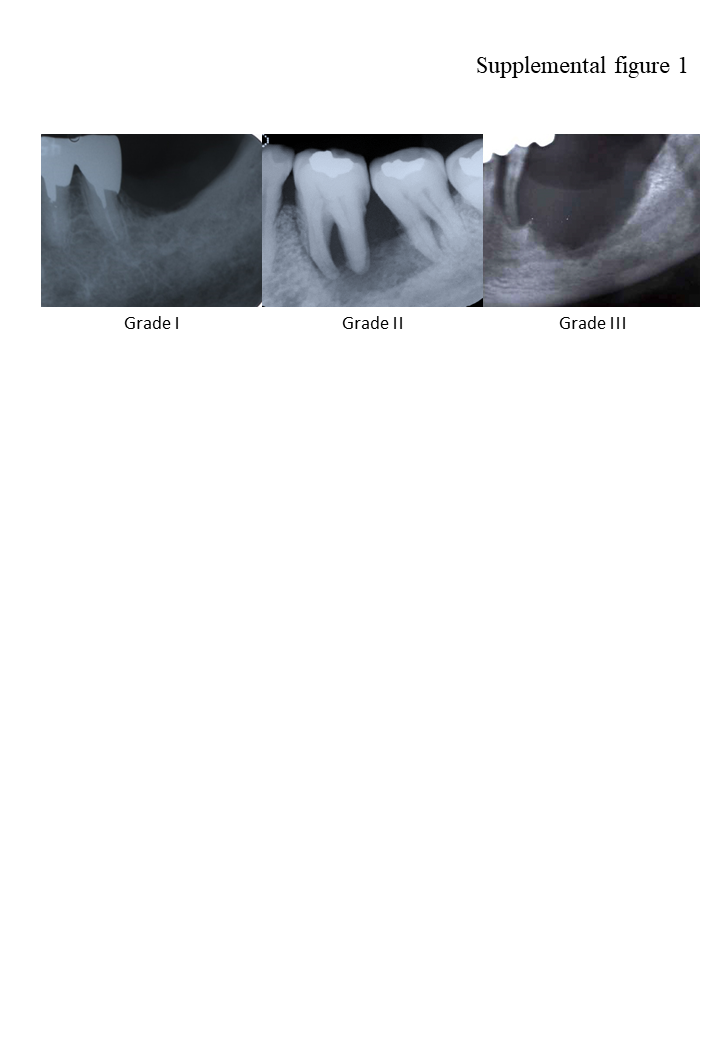

Supplement: S1 Fig — Grade I: No bone resorption or only bone erosion on the superficial surface. Grade II: Bone resorption observed within the alveolar bone. Grade III: Bone resorption involving inferior alveolar nerve / floor of maxillary sinus. (TIF) [file pone.0187092.s001.TIF]

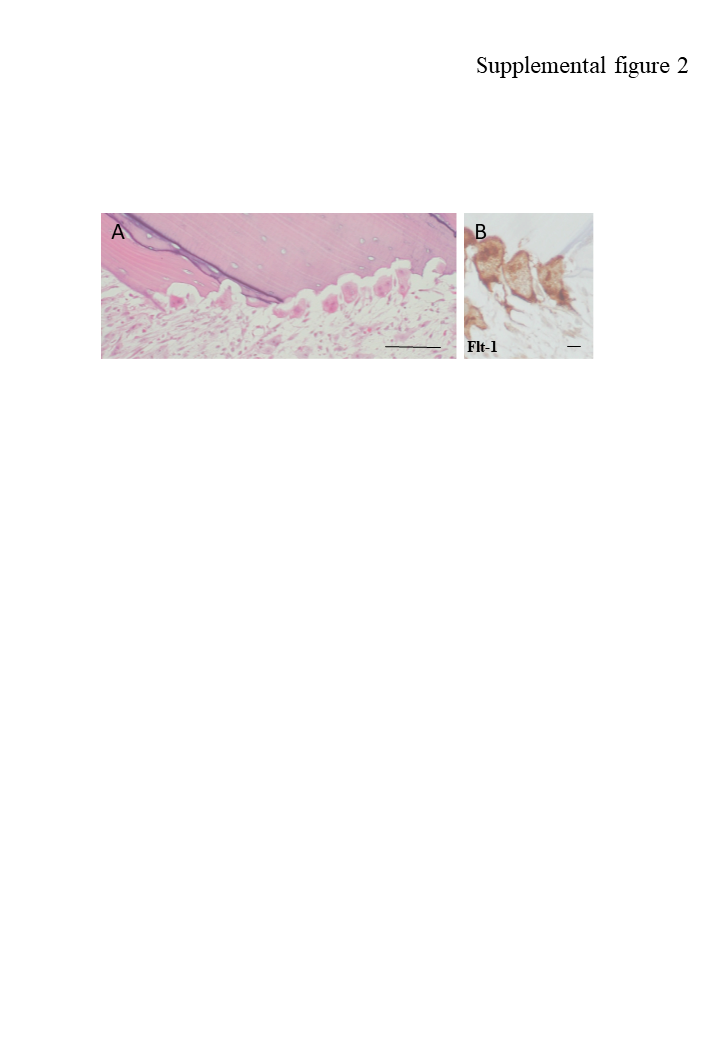

Supplement: S2 Fig — (A) Osteoclasts are seen along the irregular bone margin at the tumor/bone interface. H&E staining. Scale bar = 100 μm. (B) Osteoclasts are positively stained with Flt-1. Immunohistochemistry, Scale bar = 10 μm. (TIF) [file pone.0187092.s002.TIF]

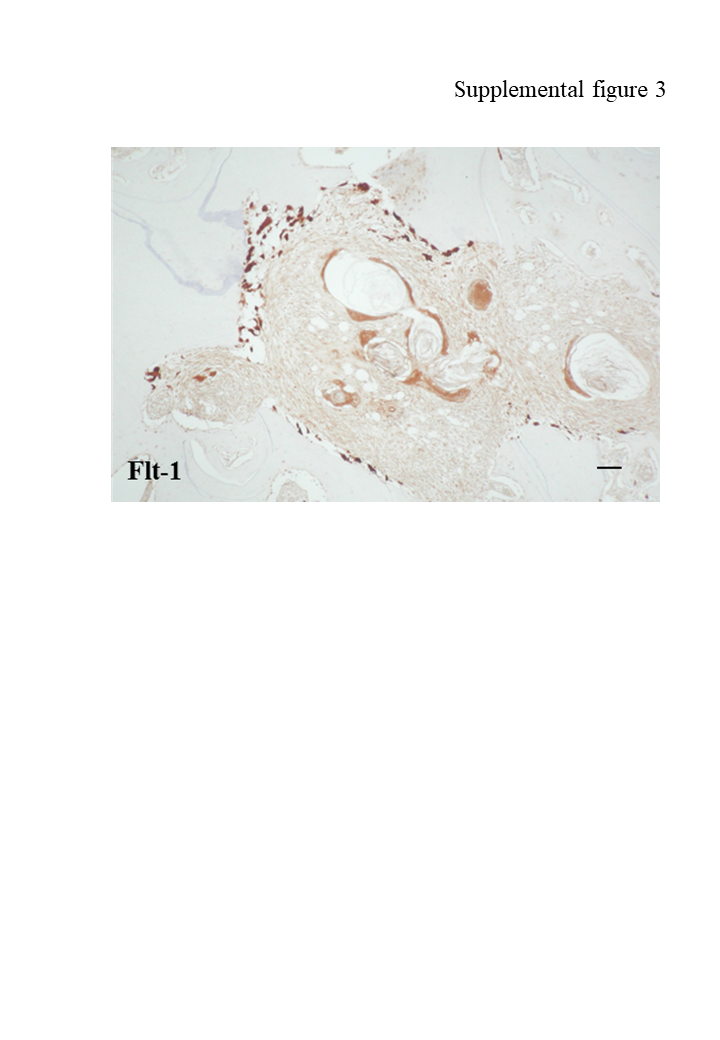

Supplement: S3 Fig — Flt-1-positive reaction was seen in osteoblasts/preosteoblasts along the bone surface as well as in OSCC cells. Immunohistochemistry, Scale bar = 100 μm. (TIF) [file pone.0187092.s003.TIF]

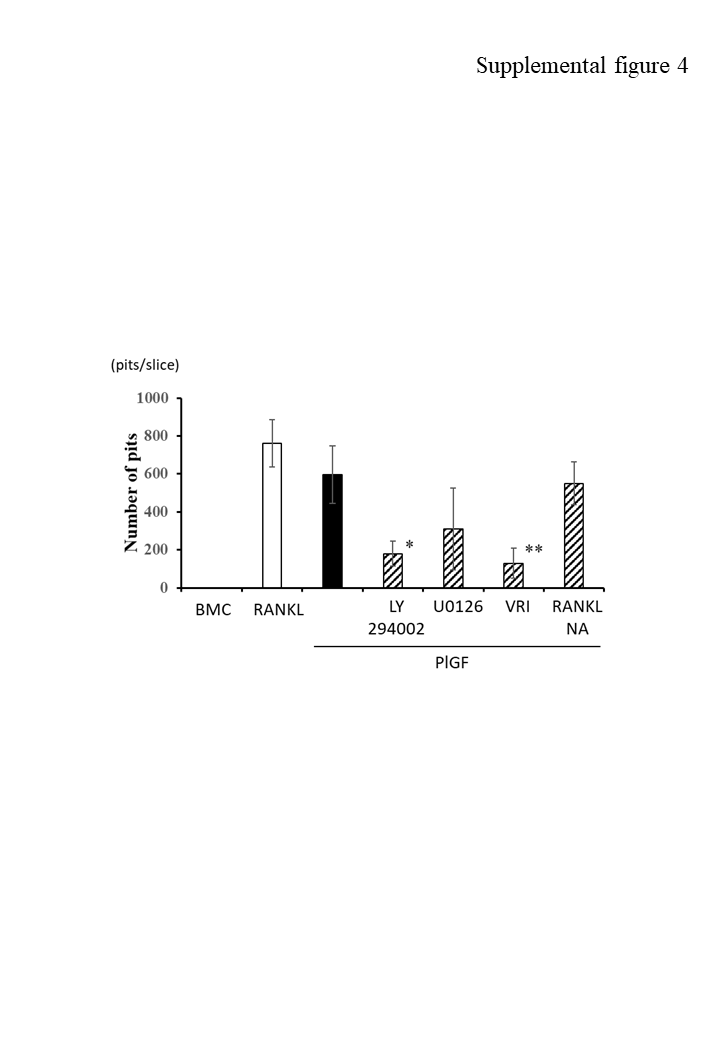

Supplement: S4 Fig — Trypsinized BMCs were plated on dentin slices in 96-well culture plates and cultured for 1 hour. The dentin slice were then transferred into 48-well culture plates and culture in α-MEM containing Flt-1-specific ligand (PlGF (10 ng/ml)) with/without Akt inhibitor (LY29400: 10 μM), ERK inhibitor (U0126: 10 μM), VEGF tyrosine kinase inhibitor II (VRI: 10 μM) or RANKL-neutralizing antibody (5 μg/ml) was performed. RANKL stimulated BMC culture on dentin slice also done as a positive control. *p<0.05, **p<0.01. (TIF) [file pone.0187092.s004.TIF]
